# Connexin-43: A possible mediator of heat stress effects on ram Sertoli cells

**Published:** 2015-06-15

**Authors:** Hossain Hassanpour, Ali Kadivar, Pejman Mirshokraei, Hassan Nazari, Azita Afzali, Maryam Badisanaye

**Affiliations:** 1 *Research Institute of Animal Embryo Technology, Shahrekord University, Shahrekord, Iran; *; 2 *Department of Clinical Science, Faculty of Veterinary Medicine, Shahrekord University, Shahrekord, Iran; *; 3 *Research Institute of Biotechnology, Shahrekord University, Shahrekord, Iran; *; 4 *Department of Clinical Science, School of Veterinary Medicine, Ferdowsi University of Mashhad, Mashhad, Iran; *; 5 *DVM Student, Department of Basic Science, Faculty of Veterinary Medicine, Shahrekord University, Shahrekord, Iran.*

**Keywords:** Connexin-43, Heat stress, Ram, Sertoli cell

## Abstract

Sertoli cells are an essential group of cells in seminiferous epithelium which provide nutritional and structural supports for spermatogenic cells via cell junctions. In this study, the gene expression of connexin-43, the most abundantly distributed gap junction protein of cells, was investigated in ram Sertoli cells under mild and severe heat stresses with real-time quantitative PCR. Sertoli cells were isolated from testes of 10 lambs. After culture and 3 passages, they were treated with mild (39 ˚C) and severe (42 ˚C) heat stress for 6 hr. The results showed a significant reduction in the percentage of live cells under severe heat stress compared to the control group (32 ˚C), (*p* <0.05). Relative quantification analysis revealed significantly higher (3.80 fold increase) values of connexin-43 transcripts in severely heat stressed group than control group (*p* <0.05). It is concluded that challenging Sertoli cells with 42 ˚C heat could threaten their survival, and overexpression of connexin-43 may cause dysfunction of Sertoli cells due to heat stress. These findings can be useful to identify the molecular mechanisms involved in adverse effects of heat stress on male reproduction and enhance our understanding of its pathogenesis.

## Introduction

Animal cells are connected by complex junctions, into tissues by special proteins. Such elaborate proteins in gap junctions, ensure and control communication and flow of materials between cells. The exchange of small molecules such as ions is crucial for the regulation of essential processes during cell differentiation and development. In addition, metabolic and electric coupling of cells, coordinated responses of coupled cells to hormones, is accomplished by exchange via these junctions.^1^ In the epithelium of seminiferous tubules, the coupling of gap junctions occurs as Sertoli-Sertoli cells, Sertoli - spermatogenic cells and spermatogenic - spermatogenic cells.^2^

Each gap junction is composed of two Connexons and each Connexon is formed of six protein subunits known as connexins.^3^ Several connexin proteins such as connexin-26 and connexin-32 have been reported between Sertoli cells and germ cells in testes.^4^ Transcripts of connexin-50 and connexin-33 have also been detected in meiotic germ cells.^5^ However, the most abundantly distributed gap junction protein is conexinx-43 in testicular cells.^6^

The importance of connexin-43 in spermatogenesis has been demonstrated in knockout mouse models. Such mice had hypotrophic testes because of severe germ cell deficiency.^7^ Grafting of the testes of connexin-43 knockout fetuses under the renal capsules of adult males resulted in germ cell deficiency in seminiferous epithelium.^8^ The critical role of connexin-43 has also been reported in human spermatogenesis, as it was reduced in infertile men with azoospermia.^9^

In the epithelium of the seminiferous tubules, sperm development occurs in contact with Sertoli cells. Cellular interactions between Sertoli cells and peritubular and spermatogenic cells are important for the maintenance and differentiation of sperms.^10^ In a specific epithelial stage, an individual Sertoli cell establishes contacts with five other Sertoli cells, as well as 50 spermatogenic cells in different stages of sperm development.^11^ This extensive coupling of diverse cell types is permitted by gap junctions in the seminiferous epithelium.

Furthermore, many connexin proteins detected in testes originate from interstitial tissue, while only connexin-33 and connexin-43 proteins have been identified in the gap junctions between Sertoli cells.^12^ In neonatal rats, an inactive form of connexin-43 is predominantly localized within the cytoplasm of Sertoli cells, whereas during the terminal differentiation of Sertoli cells in pubertal period, connexin-43 is localized in the plasma membrane as active form.^13^ In addition, it has been demonstrated that an increase in the expression of connexin-43 mediated by thyroid hormone, reduces proliferation in the Sertoli cell, suggesting that connexin-43 may regulate Sertoli cell proliferation.^14^

Since mature Sertoli cells are the primary supportive cells of seminiferous epithelium and provide an essential nutritional and structural support for the developing spermatogenic cells,^10^ any impairment in their function can significantly disturb spermatogenesis.

It has been confirmed that the high temperature as an important stress factor, could considerably influence the male fertility. In this regard, elevation of testicular temperature disturbs the function and morphology of Sertoli cells and subsequent germ cell loss and infertility.^15^ The molecular mechanisms of heat stress on Sertoli cells are poorly understood. Based on emerging literature demonstrating that connexin-43 is involved in the regulation of Sertoli cell functions, we attempted to evaluate the possible changes in connexin-43 gene expression in these cells under heat stress. Such studies offer the potential to enhance our understanding of the molecular mechanisms involved in negative effects of heat stress on male reproductive system and have clinical applications.

## Materials and Methods

Cell cultures and treatments. All materials used in this study, except those mentioned, were purchased from Sigma (Sigma-Aldrich Co., St. Louis, USA). Testes of 10 lambs (3 to 10 month-old) were collected from an abattoir, placed on ice and transferred to the laboratory within 2 hr. Sertoli cells were isolated from these testes and cultured according to Izadyar et al.^16^ This procedure with some modifications is as follow:

Testes were decapsulated and minced into small pieces. In the first stage of enzymatic digestion, testis pieces were suspended in Eagle's minimal essential medium (EMEM) + 25 mM NaHCO_3_ and incubated for 1 hr at 37 ˚C with 50 IU mL^-1^ DNase ( Roche Diagnostics, Indianapolis, USA), 1 mg mL^-1^ trypsin and 1 mg mL^-1^ type IV collagenase (Gibco, Grand Island, USA). The samples were centrifuged at 400 g for 4 min and supernatants (containing Leydig cells) were discarded. In the second stage of enzymatic digestion, the samples were suspended in Eagleʼs minimum essential medium (EMEM; Gibco, Grand Island, USA) + 25 mM NaHCO_3_ and incubated at 37 ˚C (5% CO_2_) for 45 min with DNase (50 IU mL^-1^) and type IV collagenase (1 mg mL^-1^). The centrifugation was repeated again at 60 g for 30 sec. The supernatant was isolated, transferred to a fresh tube and then was centrifuged at 400 g for 4 min. The pooled cells were incubated at 32 ˚C in EMEM supplemented with 10% fetal calf serum (FCS; Gibco, Grand Island, USA), 25 mmol NaHCO_3_, 2 mmol L-glutamine, 1% non-essential amino acids, 200 IU mL-1penicillin, 0.2 mg mL-1 streptomycin and 15 mmol N-(2-hydroxyethyl) piperazine-N'-ethane-sulfonic acid (HEPES; Sigma Chemical Co., St. Louis, USA). After 24 hr, cells in the supernatant fluid were discarded. At this time the round to cuboidal cells that adhere to the Petri dish are Sertoli cells. The culture process is continued with renewing the culture medium. After three passages, Sertoli cells with 70% confluency were subdivided into three groups: 1) control group, cell incubation at 32 ˚C for 6 hr, 2) mild heat stress group, cell incubation at 39 ˚C for 6 hr, and 3) severe heat stress group, cell incubation at 42 ˚C for 6 hr.

Cell viability assay. Trypan blue dye exclusion was used to determine the percentage of viable cells. As a non-vital dye, trypan blue is excluded from living cells, however, stains dead cells. Equal volumes (200 µL) of cell suspension and filter sterilized 0.4% (w/v) trypan blue in PBS were mixed, and cells were counted under a light microscope using hemocytometer (Kayagaki, Tokyo, Japan). This test was carried out on all experimental groups.

RNA extraction and cDNA synthesis of Sertoli cells. Selected plates for RNA extraction had 70% of cell confluency and 4.5 to 5 × 10^6^ cells per plate (based on counting with hemocytometer). Total RNA isolation was carried out on Sertoli cells according to the acid guanidinium thiocyanate-phenol-chloroform single-step extraction protocol.^21^ Treatment of total RNA with RNase-free DNase (SinaClon BioScience Co., Karaj, Iran) was performed to avoid amplification of contaminating genomic DNA. The quality and integrity of the purified RNA was controlled by measurement of the A260/A280 nm ratio and by agarose gel electrophoresis. Only RNA samples showing integrity of the RNA by electrophoresis and exhibiting an A260/A280 ratio > 1.90 were used for synthesis of cDNA. Total RNA was reverse transcribed into cDNA using M-MLV reverse transcriptase (SinaClon BioScience Co., Karaj, Iran) as described by Hassanpour et al.^17^ The reverse transcription mixure was heated to 75 ˚C for 15 min to denature the RNA, and then stored at – 20 ˚C.

Real-time quantitative PCR analysis. The levels of connexin-43 transcripts were determined by real-time reverse transcriptase (RT)-PCR using Eva-Green chemistry (SinaClon BioScience Co., Karaj, Iran). Glyceraldehyde-3-phosphate dehydrogenase (GAPDH) was selected as a house-keeping gene to normalize input load of cDNA between samples. Specific primers for connexin-43 and GAPDH were designed using primer BLAST.^18^ The nucleotide sequences of the primer pair selected for GAPDH were as follow: forward, 5΄-TGGCAAAGTGGACATCGTTG-3΄ and reverse, 5΄ TGGCGTGGACAGTGGTCATAAGTC-3΄ with amplified product of expected 467 bp. The nucleotide sequences of the primer pair selected for connexin-43 were as follow: forward, 5΄-TCGTGTCGTTGGTGTCTCTTG-3΄ and reverse, 5΄-GAGGAGCAGCCATTGAAATAAGC-3΄. These primers yielded a 200-bp product. Real-time quantitative PCR (RT-qPCR) analysis was performed on Rotor-Gene Q 6000 System (US) using the Titan Hot Taq Eva-Green Ready Mix (SinaClon BioScience Co., Karaj, Iran). A volume of 1 µL cDNA was added to the mixture of 0.5 µM of each specific primer and 4 µL of Titan Hot Taq Eva-Green Ready Mix (SinaClon BioScience Co., Karaj, Iran) in a total volume of 20 µL. An aliquot of each reaction mixture was subjected to electrophoresis in 1.5% agarose gel and stained with 0.5 μg mL^-1^ ethidium bromide. The relative quantification of connexin-43 transcripts was determined among the groups. Reaction condition was 95 ˚C for 10 min, 40 cycles of 95 ˚C for 40 sec, 63 ˚C for 45 sec and 72 ˚C for 30 sec. The PCR amplification was performed in triplicate for each sample with connexin-43 and GAPDH.

Data from a standard curve were used to calculate PCR amplification efficiencies of connexin-43 and GAPDH. The cycle threshold (CT) values of the target gene (Connexin-43) were normalized to those of the reference gene (GAPDH), and the relative quantification was performed according to the ΔΔCT model,^19^ using Rotor-Gene Q software, (Version 2.0.2; Qiagen, Venlo, The Netherlands). To ensure product homogeneity, the melting curve analysis was performed after the real time PCR procedure. The fluorescence signals were recorded continuously during temperature ramp.

Statistical analysis. Differences between experimental group means were analyzed through one-way analysis of variance (ANOVA) of SPSS (Version 16; SPSS Inc., Chicago, USA) followed by Duncan’s multiple range tests. All results are shown as mean ± SEM and differences were considered significant at p < 0.05.

## Results

**Fig. 1:**
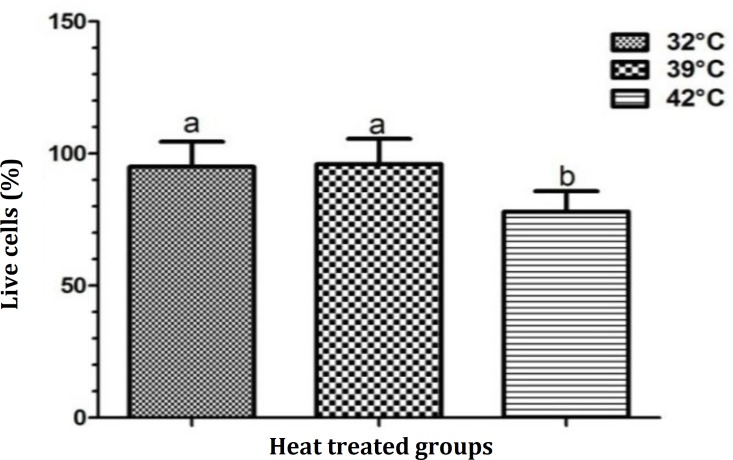
The percentage of live cells in different heat-treated groups of Sertoli cells. ^a,b^ Different superscripts indicate statistical significant difference at p < 0.05

The results of cell viability assay are presented in Fig. 1. Severe heat stress caused a significant reduction in the percentage of live cells compared to the control group.

**Fig. 2:**
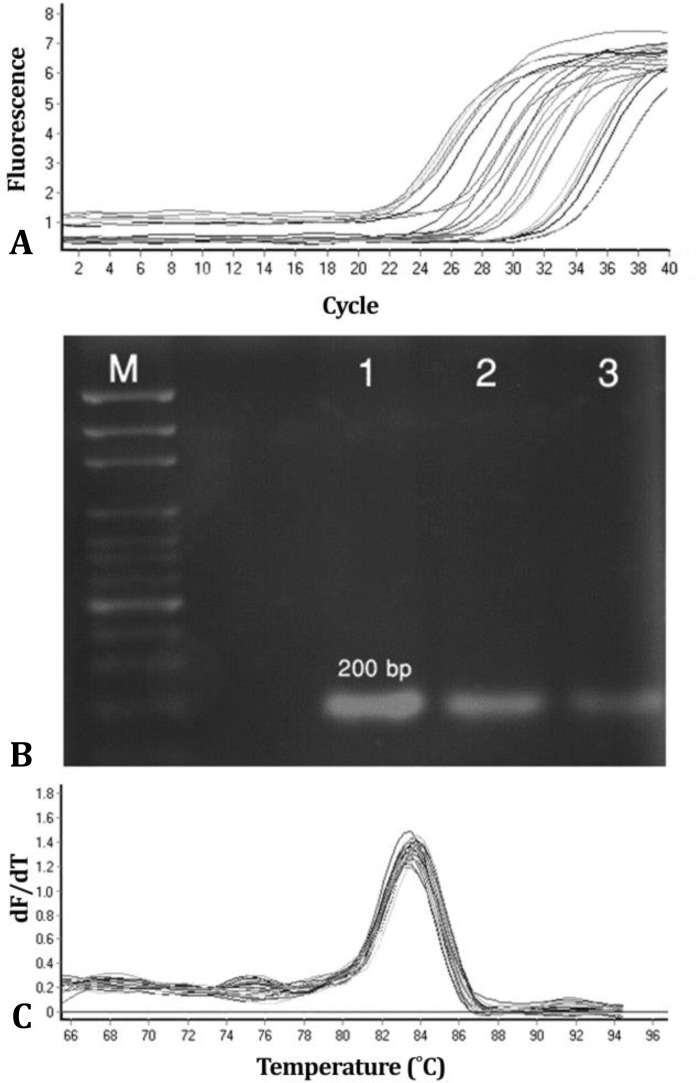
A) Results of electrophoresis for PCR products from testing samples with connexin-43 primer; M: 100 bp marker; Lane 1: 42 ˚C heat stress group; Lane 2: 39 ˚C heat stress group; Lane 3: Control group. Connexin-43 amplification plot; B) Dissociation curves for connexin-43 amplicons; C) The negative derivative of fluorescence versus temperature (dF/dT) is plotted against temperature. The curves have single peaks, suggesting that only specific PCR products were generated with these sets of primers

**Fig. 3:**
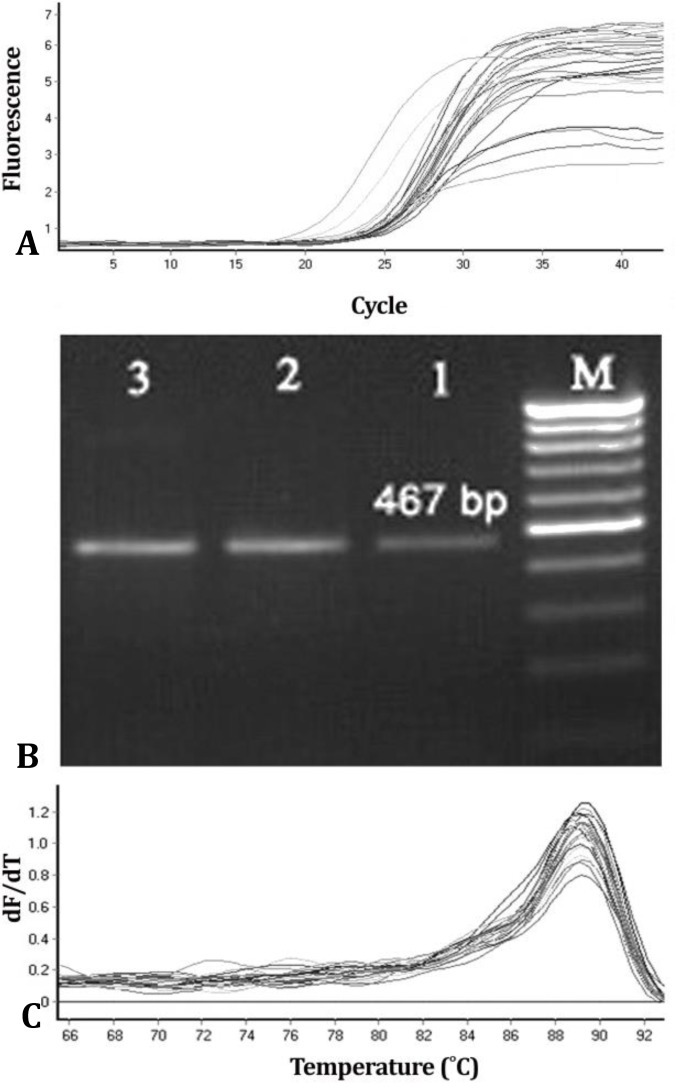
A) Results of electrophoresis for PCR products from testing samples with glyceraldehyde-3-phosphate dehydrogenase (GAPDH) primer; M: 100 bp marker; Lane 1: 42 ˚C heat stress group; Lane 2: 39 ˚C heat stress group; Lane 3: Control group. GAPDH amplification plot; B) Dissociation curves for GAPDH amplicons; C) The negative derivative of fluorescence versus temperature (dF/dT) is plotted against temperature. The curves have single peaks, suggesting that only specific PCR products were generated with these sets of primers

There was amplification in all samples for GAPDH and connexin-43/qPCR, demonstrating 100% efficiency of cDNA extraction (Figs. 2A and 3A). Moreover, the amplification efficiencies for GAPDH and connexin-43 were 1.87 and 1.92, respectively.

 The extracted cDNA from Sertoli cells showed one band at 200 bp after amplification with connexin-43 primer and at 467 bp after amplification with GAPDH primer (Figs. 2B and 3B). Data were normalized as ΔCT (difference between mean CT value of connexin-43 and mean CT value of GAPDH), and expressed as fold differences between three heat treatment groups. 

**Fig. 4:**
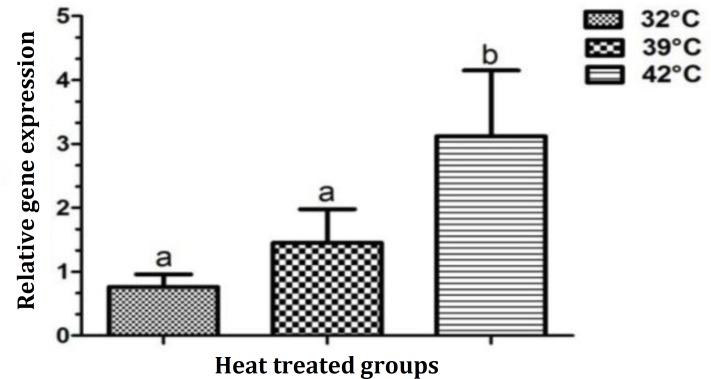
Relative expression of connexin-43 gene in different heat treated groups of Sertoli cells. ^a,b^ Different superscripts indicate statistical significant difference at p < 0.05

As seen in Figure 4, the values of connexin-43 transcripts were significantly higher (showing 3.80 fold increase) in severe heat stress group than control group (2.92 ± 0.98 versus 0.76 ± 0.20). The amount of connexin-43 gene expression was also higher in mild heat stress group than control (1.53 ± 0.56 versus 0.76 ± 0.20), however, this difference was not statistically significant (Table 1).

Homogeneity of the accumulated PCR products was confirmed in the assays by dissociation curves, which showed only single sharp peaks (Figs. 2C and 3C).

**Table 1:** Relative expression of connexin-43 gene (Mean ± SEM) and fold change rate of expression relative to control group in mild and severe heat stress groups.

**Groups**	**Connexin-43 relative expression**	**Fold change rate relative to control (32 ˚C)**
**Control (32 ˚C)**	0.76 ± 0.20	-
**Mild heat stress (39 ˚C)**	1.53 ± 0.56	1.99
**Severe heat stress (42 ˚C)**	2.92 ± 0.98	3.80

## Discussion

In most mammals, normal spermatogenesis occurs in about 2 to 8 ˚C lower than the body temperature,^20^ and it has been shown that hyperthermia can induce testicular germ cell apoptosis, and consequently azoospermia or oligospermia in rodents.^21^ Germ cells are largely dependent on Sertoli cells for structural and nutritional support, and the number of germ cells in the seminiferous tubules is influenced by the number of Sertoli cells.^11^ Also, the tight junctions between the adjacent Sertoli cells constitute the blood-testis barrier, providing protective environment for germ cell development.^22^ Moreover, effects of FSH and testosterone on spermatogenesis are regulated by Sertoli cells.^23^

Some reports have demonstrated that hyperthermia could alter functions of Sertoli cells. The study of Zhang etal. indicated that exposure of adult monkey and rat Sertoli cells to 43 ˚C heat stress induced re-expression of liver receptor homolog-1 and cytokeratin-18 in the differentiated cells. This may represent a dedifferentiated feature of adult Sertoli cells.^24^ However, no evidence of changes was reported in the gap junctions of Sertoli cells following heat treatment. In this study, we have demonstrated for the first time that 42 ˚C heat treatment of Sertoli cells could induce overexpression of connexin-43.

Several studies have shown that tissue homeostasis is influenced by connexin proteins. In most cases, changes in connexin expression are independent of its channel forming capabilities. Overexpression of connexin was found to initiate cell cycle arrest without influencing connexin channel forming activity. In human glioblastoma cells, forced expression of connexin-43 enhances the apoptotic response to chemotherapeutic drugs without increasing gap junction coupling.^25^ These studies suggest that there are distinct roles for connexin-43 in the cell death and cell survival.

According to the results of present study, there is an overexpression of connexin-43 under heat stress condition, and this induction coincides with elevation of cell death under such circumstances. The underlying mechanisms of connexin protein contributions to cell death have not been completely identified thus far. One possible mechanism for the involvement of connexins in cell death pathways could be through direct interaction with apoptotic factors. In this regards, connexin-43 has been co-localized with pro-apoptotic proteins such as Bak and Bax in the cytoplasm of human breast and colorectal cancer cells.^26^ Other possible mechanism could be its involvement in the control of cell death-related gene expression. In fact, a number of studies have reported interference of connexin gene expression along with changes in the expression of many apoptotic factors.^27^ For instance, Walker et al. found that the expression of a wide spectrum of apoptotic genes (including Bax, Bok, Bid, caspase-6 and caspase-9) were altered in the connexin-43 knockout mice.^28^ Connexins are proposed to have the feature of modulating gene expression. It was observed that connexins or the specific parts of these molecules (e.g., the C-terminal region) were resided in the nuclear compartment and they interacted with transcriptional regulators and/or mediators of crucial signaling pathways.^29^ Indeed, some of connexin binding partners such as β-catenin are known as regulators of gene expression.^29^ This regulatory molecule is a key player in Wnt signaling^30^ which forms a complex with the T-cell factor in the cell nucleus that facilitates the transcription of a number of apoptotic genes including Bcl-2.188 and Bcl-xL187. According to Sharrow et al. that tumor necrosis factor α not only increased degradation of connexin-43 protein but also augmented the connexin-43 mRNA content in human osteosarcoma cells. They suggested that the enhanced connexin-43 gene transcription might represent a reflexive response to apoptosis.^31^

Testicular tissues particularly Sertoli cells are sensitive to temperature changes and to preserve their exposure to heat stress induces a chain of protective responses, including modification of communication and flow of materials between cells. These modified connections between cells in the heat stress harmfully causes more flow of many apoptotic factors which enhance the apoptosis and cell death. Thus, challenging Sertoli cells with 42 ˚C heat endangers cell survival due to overexpression of connexin-43. These findings can shed further light on the molecular mechanisms involved in deleterious effects of heat stress on male reproductive system and provide insight for additional experimentation.
